# Endothelial-specific CXCL12 regulates neovascularization during tissue repair and tumor progression

**DOI:** 10.1096/fj.202401307R

**Published:** 2024-12-13

**Authors:** Andrew C. Hostler, William W. Hahn, Michael S. Hu, Robert Rennert, Katharina S. Fischer, Janos A. Barrera, Dominik Duscher, Michael Januszyk, Dominic Henn, Dharshan Sivaraj, Jonathan P. Yasmeh, Hudson C. Kussie, Maia B. Granoski, Jagannath Padmanabhan, Ivan N. Vial, Johannes Riegler, Joseph C. Wu, Michael T. Longaker, Kellen Chen, Zeshaan N. Maan, Geoffrey C. Gurtner

**Affiliations:** 1 Department of Surgery, The University of Arizona College of Medicine, Tucson, Arizona, USA; 2 Department of Surgery, Division of Plastic and Reconstructive Surgery, Stanford University School of Medicine, Palo Alto, California, USA; 3 Stanford Cardiovascular Institute, Stanford University School of Medicine, Stanford, California, USA

**Keywords:** tissue repair, wound healing, neovascularization, CXCL12, molecular biology, chemokines, cytokines, cancer, endothelial cells

## Abstract

C-X-C motif chemokine ligand 12 (CXCL12; Stromal Cell-Derived Factor 1 [SDF-1]), most notably known for its role in embryogenesis and hematopoiesis, has been implicated in tumor pathophysiology and neovascularization. However, its cell-specific role and mechanism of action have not been well characterized. Previous work by our group has demonstrated that hypoxia-inducible factor (HIF)-1 modulates downstream CXCL12 expression following ischemic tissue injury. By utilizing a conditional CXCL12 knockout murine model, we demonstrate that endothelial-specific deletion of CXCL12 (eKO) modulates ischemic tissue survival, altering tissue repair and tumor progression without affecting embryogenesis and morphogenesis. Loss of endothelial-specific CXCL12 disrupts critical endothelial–fibroblast crosstalk necessary for stromal growth and vascularization. Using murine parabiosis with novel transcriptomic technologies, we demonstrate that endothelial-specific CXCL12 signaling results in downstream recruitment of non-inflammatory circulating cells, defined by neovascularization modulating genes. These findings indicate an essential role for endothelial-specific CXCL12 expression during the neovascular response in tissue injury and tumor progression.

## Graphical Abstract

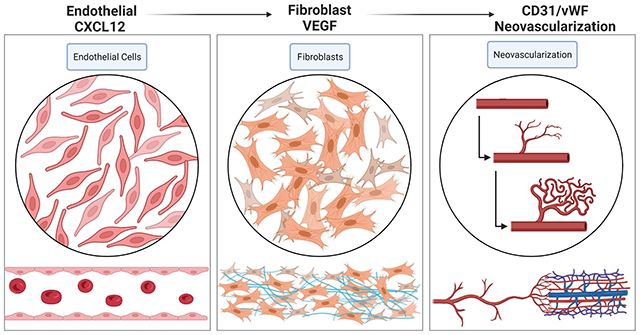

## 1 | INTRODUCTION

Neovascularization, the assembly of new blood vessels, plays a crucial role during tissue repair and regeneration. The process of neovascularization influences functional outcomes after injury to cardiac tissue, neural tissue, and other periphery tissues in response to ischemia, inflammation, genetic aberrations, and mechanical stimulation.^1–3^ The complex biological processes governing neovascularization and tissue repair are exploited by tumor cells^4,5^ which typically exist in a setting of relative ischemia.^6^ Tumorigenesis, progression, and survival critically rely on the surrounding stromal microenvironment.^7,8^ Uninhibited cell division and proliferation result in the tumor surpassing the oxygen constraints of the preexisting, native tissue vasculature resulting in a state of perpetual hypoxia. While numerous studies have explored the importance of a vascularized stroma to both physiologic tissue repair and pathologic tumor development,^9^ an insufficient understanding of the underlying molecular mechanisms has limited the development of efficacious, targeted therapies.

Previous work by our group and others has described the pivotal role of C-X-C motif chemokine ligand 12 (CXCL12) in the recruitment of circulating cells to hypoxic tissue, following stabilization of hypoxia-inducible factor (HIF)-1, a cellular-hypoxia adaptation transcriptional factor,^10^ and regulation of stem cell microenvironments.^11,12^ Interestingly, CXCL12 is also widely expressed in several human tumors and has been identified in the pathogenesis of tumorigenesis and survival.^13–15^ While these reports suggest a critical role for CXCL12 during tissue repair and tumor progression, the mechanisms underlying CXCL12’s influence on these hypoxic-related processes remain poorly understood. Thus, we hypothesize that interrogating the role of CXCL12 in neovascularization will provide an opportunity to unravel the complex molecular mechanisms underpinning tumor biology, tumor pathophysiology, and tissue repair.

Herein, our group engineered a conditional CXCL12 knockout mouse to identify a critical role for endothelial-specific CXCL12 expression during neovascularization and tumor progression. Knockout of endothelial-specific CXCL12 reduced wound healing and ischemic tissue survival. Surprisingly, loss of endothelial-specific CXCL12 expression completely abrogated tumor growth despite elevated stromal CXCL12 expression by tumor microenvironment-associated cell populations. We demonstrate that loss of endothelial-specific CXCL12 inhibits fibroblast proliferation, survival, and expression of angiogenic cytokines, clarifying the importance of endothelial-fibroblast crosstalk in this process. Finally, by utilizing single-cell gene expression analyses in combination with a parabiosis model, we identified a non-inflammatory progenitor cell subpopulation exclusively recruited to tissue by endothelial-specific CXCL12 signaling. Our findings reveal a crucial role for endothelial-derived CXCL12 in both tissue repair and tumor progression pathogenesis, demonstrating a paracrine mechanism governing vascularized stromal patterning.

## 2 | MATERIALS AND METHODS

### 2.1 | Study approval

Animal experiments were approved by the Stanford University Administrative Panel on Laboratory Animal Care (APLAC) (Stanford, CA, USA).

### 2.2 | Animal studies

Studies using murine models were initially carried out using *n* = 5 mice and repeated unless specifically denoted. Throughout all animal studies, female mice 14 ± 2 weeks old were used. All animal experiments were compliant with ethical regulations.

### 2.3 | Mice

All transgenic murine strains were backcrossed onto a C57BL/6J background (Jackson Laboratories, Bar Harbor, ME). *Rosa–CreER* (B6.129-Gt[ROSA]26Sortm1 [cre/ERT2]Tyj/J; Jackson Strain #008463), *Tie2–Cre* (B6.Cg-Tg[Tek-cre]1Ywa/J; Jackson Strain #008863), and *Col1a2-CreER* (B6.Cg-Tg[Col1a2-cre/ERT,-ALPP]7 Cpd/2J; Jackson Strain #029567) mice were obtained from the Jackson Laboratory. Similarly, GFP-positive ([*C57BL/6-TgCAG-EGFP]1Osb/J*; *Jackson Strain*#003291) mice were obtained from Jackson Laboratory. Mice were maintained under standard pathogen-free conditions according to APLAC policy and procedure.

### 2.4 | Generation of *Cxcl12^loxP/loxP^* mice

**FIGURE 1:**
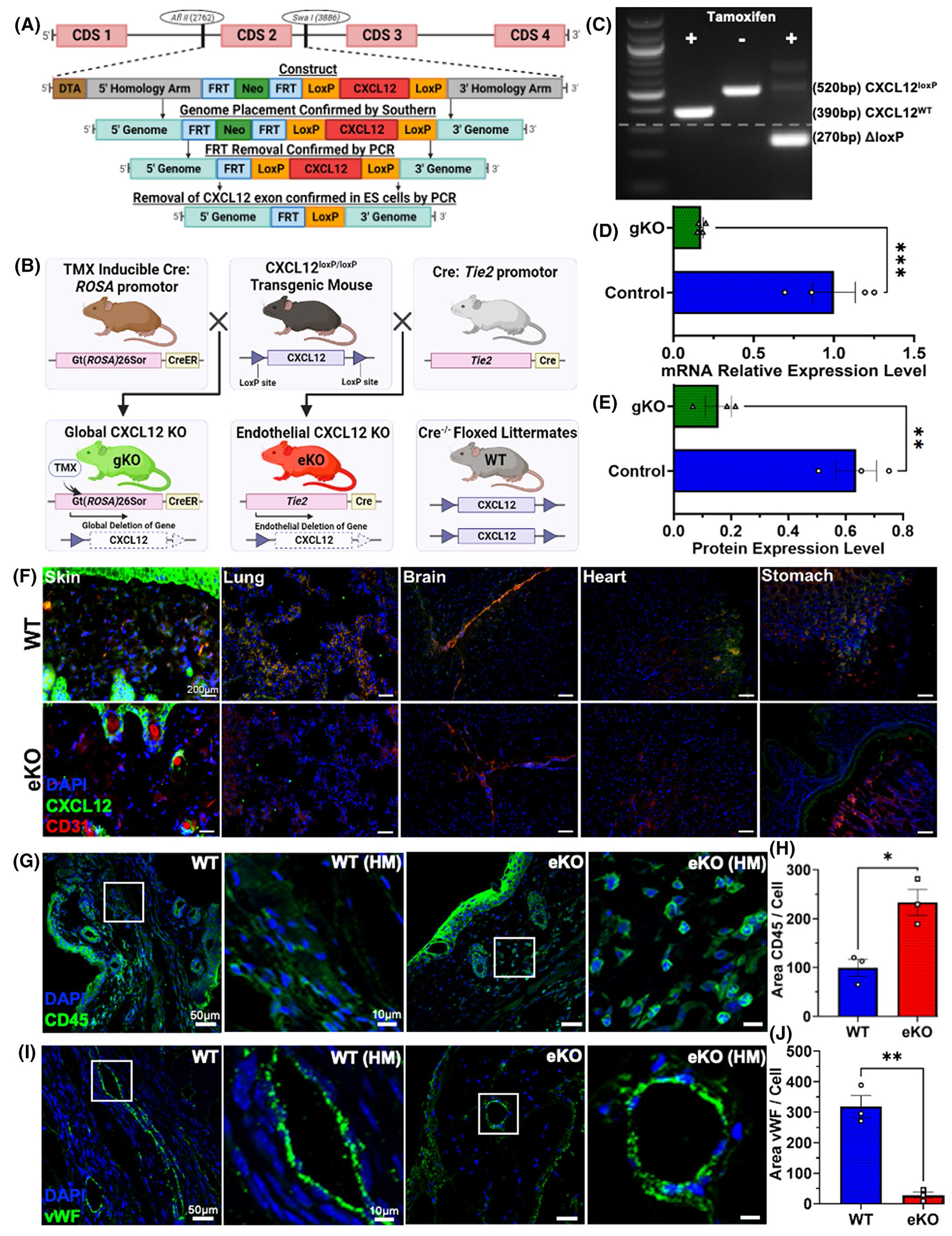
Development and validation of both global and endothelial-specific CXCL12 knockout murine models. (A) This illustration depicts the murine *Cxcl12* locus our group utilized for homologous recombination in embryonic stem cells to transgenically engineer a floxed *Cxcl12* allele. Cre-recombinase transgenic mice were crossed with our group’s *Cxcl12* floxed mice to generate novel tissue-specific knockout progeny specifically for this study. (B) The illustration reflects the breeding strategy to generate our group’s novel tamoxifen-inducible global CXCL12 knockout (gKO) and endothelial-cell specific CXCL12 knockout (eKO) mice. Floxed littermates that were negative for Cre (e.g., *Cxcl12*^*loxP/loxP*^) were used as our control (WT) group. (C) PCR validation of the presence and recombination of the floxed allele upon exposure of gKO mice to tamoxifen. (D) qRT-PCR of *Cxcl12* expression in the skin of tamoxifen-induced gKO mice compared to control (***p = .0008, WT: *n* = 4, gKO: *n* = 4). (E) ELISA for CXCL12 expression levels in the skin of tamoxifen-induced gKO mice compared to control (**p = .0047, WT: *n* = 3, gKO: *n* = 3). (F) Immunofluorescent staining of eKO and WT tissues (skin, lung brain, heart, and stomach) showing CXCL12 (green) and CD31 (red) co-localized expression in WT and not eKO mice. (G, H) Immunofluorescent staining and analysis of POD7 wound tissue for CD45 (green) marking hematopoietic cells (*p = .0137, WT: *n* = 3, gKO: *n* = 3). (I, J) Immunofluorescent staining and analysis of POD7 wound tissue for vWF marking vasculature (*p = .0015, WT: *n* = 3, eKO: *n* = 3). Statistical analysis was performed using an unpaired, two-tailed *t*-test (D, E, H, J). Each datapoint represents an independent subject (D, E) or independent wound (H, J). All data are presented as mean ± SEM. Representative images are shown from similar images across all subjects or wounds. Images (F) were obtained with a Zeiss Axioplan 2 fluorescence microscope, magnification ×20, scale bar 200 μm. Images (G, I) were obtained with a Zeiss LSM880 Inverted confocal microscope, magnification ×20, scale bar 50 μm, and 10 μm for high magnification (HM).

A floxed allele of *Cxcl12* was generated by inserting *LoxP* sites flanking exon 2 of *Cxcl12*. FRT sites were inserted flanking the neomycin selection cassette (Figure 1A). Generation of targeted embryonic stem cells and blastocyst injections were performed as previously characterized.^12^ Excision of the neomycin cassette was accomplished through FLP-FRT recombination. Mice were genotyped using PCR primers: *Cxcl12^loxP^* forward, 5′-ACCCATAAATTGAAACATTTGG-3′; *Cxcl12^loxP^* reverse, 5′-TTCTACCACCTGCAGTTTTCC-3′; *Cxcl12^loxP^* recombined, 5′-GGTAAATTTATCGAATTCCGAA-3′.

### 2.5 | Murine excisional wound model

Splinted, excisional wounds were created as previously described.^16^ Briefly, using a 6-mm biopsy punch (Integra Miltex, Plainsboro, NJ), two 6-mm full-thickness wounds were created on the shaved dorsum of anesthetized mice. In parabiotic pairs, the recipient mouse was only wounded on the non-parabiosed side. A donut-shaped silicone splint (10-mm diameter) was centered on the wound and secured to the skin using an immediate-bonding adhesive (Krazy Glue; Elmer’s Inc., Columbus, Ohio) and 6–0 nylon sutures (Ethicon Inc. Somerville, NJ) to prevent wound contraction, promoting deposition of granulation tissue to promote human-like wound healing conditions. All wounds were covered using an occlusive dressing (Tegaderm; 3M, St. Paul, MN). Following surgery, the mice were placed on warming pads and allowed to fully recover from anesthesia before being returned to the institutional animal facility. Wound dressings were removed and changed every 2 days. Digital photographs were taken every 2 days. Wounds were healed by secondary intention from the formation of granulation tissue at the base of the wound and re-epithelialization until all edges of the epithelial tissue had been approximated, resulting in complete wound closure. The wound area was measured using ImageJ software (NIH). All measurements were performed by a blinded observer. Wound tissue was harvested on post-wounding day 7 and once wounds were completely healed using an 8 mm punch biopsy.

### 2.6 | Murine ischemic skin flap model

A reproducible model of graded soft tissue ischemia was created on the dorsum of mice as previously described.^10^ Briefly, a full-thickness (epidermis, dermis, and underlying adipose tissue) 3-sided peninsular flap (1.25 × 2.5 cm) was created on the shaved dorsum. The flap was elevated from the underlying muscular bed and a 0.13 mm thick silicone sheet was inserted to separate the skin from the underlying tissue. The skin flap was sutured back into place with 6–0 nylon sutures. This model creates a gradient of increasing ischemia from proximal to distal. Digital photographs were taken at regular intervals and the necrosed area was measured using ImageJ software (NIH). All measurements were performed by a blinded observer.

### 2.7 | Murine parabiosis model

Parabiosis surgeries were carried out as previously described.^17,18^ In brief, GFP-positive (*n* = 10) “donor” and either control (*n* = 5) or eKO (*n* = 5) “recipient” mice were utilized for this experimental technique. Mice were anesthetized with controlled isoflurane and provided buprenorphine for analgesia. Mice were placed on thermoregulating heating pads to prevent hypothermia and ophthalmic ointment was applied to prevent ophthalmologic complications. The corresponding fur located on the lateral flank of each mouse was carefully shaved. The skin was disinfected with both betadine solution (10% povidone-iodine) and 70% ethanol. Matching skin incisions were made from the olecranon to the knee joint of each mouse. The skin edges were undermined to create about 1 cm of free skin. 6–0 nylon sutures were used to approximate the dorsal and ventral skin. The skin was oversewn to protect the suture line. Following surgery, mice were placed on warming pads and allowed to fully recover from anesthesia before being returned to the institutional animal facility. Following surgery, buprenorphine was used for analgesia by subcutaneous injection every 8–12 h for 48 h post-operatively. After three weeks, cross-circulation was confirmed using fluorescent microscopy and FACS analysis (Figure 4A).

### 2.8 | Murine tumor model

A 1 cm incision was created on the right flank of shaved and anesthetized control, eKO, and fKO mice. A 1 × 1 cm subcutaneous pocket was created and a hydrogel seeded with 2.5 × 10^5^ B16-F10 melanoma cells (ATCC CRL-6475) was implanted and the incision closed with 6–0 nylon suture as indicated in prior work.^19^ Length (*L*), width (*W*), and height (*H*) measurements were taken using digital calipers to calculate tumor volume (*V*) using the formula: *V* = 0.5 × (*L* × *W* × *H*). Murine tumor tissue was weighed on the day of explanation to measure tumor mass (*M*). Density (*D*) was calculated using the formula: *D* = *M* (grams[g])/*V* (centimeters cubed [cm^3^]). Tumor sizes were measured by the same, blinded observer every other day until mice were euthanized at 4 weeks.

### 2.9 | Blood and skin analysis

Mononuclear cells from blood were obtained from the buffy coat layer following Ficoll–Paque density centrifugation.^20^ Skin samples, including wounded tissue and ischemic skin, were digested and cells isolated as previously described.^21^

### 2.10 | Histology and tissue analysis

For tissue fixation, tissues were placed in 4% paraformaldehyde (PFA) for 12–16 h at 4°C. Samples were prepared for embedding by soaking in 30% sucrose in PBS at 4°C for 24 h. Samples were removed from the sucrose solution and tissue blocks were prepared by embedding in Tissue Tek O.C.T (Sakura Finetek) on dry ice. Frozen blocks were mounted on a MicroM HM550 cryostat (MICROM International GmbH) and 5–8-micron thick sections were transferred to Superfrost/Plus adhesive slides (Fisher & Company, Inc.). For hematoxylin and eosin (H&E) staining, standardized protocols were used without modifications. Sections were visualized using Leica DM4000B microscope (Leica Microsystems).

### 2.11 | Immunofluorescence

Immunostaining on frozen sections was performed using the following primary antibodies: CD31 (Abcam 28364), CXCL12 (Invitrogen PA5-89116), VEGF (Abcam 52917), PDGFRA (Invitrogen 14-1401-82), vWF (Invitrogen PA5-16634), and FGF-2 (Abcam 8880). Briefly, slides were fixed in cold acetone (−20°C), and then blocked for 1 h in 5% goat serum at room temperature followed by incubation with primary antibody for 12–16 h at 4°C. Slides were then incubated for 1 h with goat anti-rabbit Alexa Fluor 488 conjugate (Invitrogen A-11034), Alexa Fluor 647 conjugate (Invitrogen A-21247), or 594 conjugate (Invitrogen A-11037). A Zeiss Axioplan 2 fluorescence microscope and Zeiss LSM880 inverted confocal microscope were used to visualize and image the slides (Carl Zeiss, Inc., Thornwood, NY). A quantification of fluorescence was performed by a blinded observer using ImageJ software (NIH).

### 2.12 | Quantification of immunofluorescent staining

Using a MATLAB code adapted by our co-corresponding author’s (K.C.)^22,23^ previous image analysis, we quantified immunofluorescent staining. Immunofluorescence images were divided into their respective RGB channels. To determine the area covered by individual color channels, they were then converted to binary. DAPI, corresponding to the blue channel, was converted to binary using imbinarize. An automated, image-specific threshold was used to optimize the number of DAPI cells counted within the image. The actual protein stains, corresponding to the red and green channels, were converted to binary with a ~0.3 consistent threshold for all images of the same stain, ensuring that the quantification of the stain area would be unbiased. To normalize the areas of the red and green stains, we divided them by the number of cells, as calculated by the number of DAPI nuclei above a 15-pixel size threshold. Confocal microscopy images were 3D reconstructed using Imaris 7.2 (Oxford Instruments, Abingdon, UK).

### 2.13 | Flow cytometry

All flow cytometry analysis was performed on dissociated wound tissue or blood. Cells were stained by standard protocols with the following fluorescently conjugated antibodies (eBiosciences unless otherwise noted). Lineage analysis was assessed using R-Phycoerythrin (PE)-Cy5-conjugated Ly6C/G (RB6-8C5, Gr-1, myeloid), CD45R (RA3-6B2, B220, B lymphocytes), TER119, CD4, CD8, and CD11b. Cells not stained with these antibodies were incubated with the proper isotype controls or left unstained. Cells were resuspended in FACS buffer and DAPI prior to FACS analysis on a FACSAria II. At least 50 000 events were recorded per sample. Data were analyzed using FlowJo digital fluorescence-activated cell sorting software by a single blinded evaluator (Tree Star Inc., Ashland, OR).

### 2.14 | Quantitative reverse-transcription PCR

RNA was isolated from explanted POD 7 eKO and WT wound tissue using an RNeasy Mini Kit (Qiagen, Hilden, Germany) according to the manufacturer’s instructions. Reverse transcription was performed with 500 ng RNA using the SuperScript III First-Strand Synthesis System (Invitrogen, Carlsbad, CA). qRT-PCR was carried out using TaqMan^®^ Assays-on-Demand^™^ Gene Expression Products from Applied Biosystems (Foster City, CA, USA): *Cxcl12*, assay ID Mm00445552_m1; *Vegfα*, assay ID Mm01281447_m1; *Fgf2*, assay ID Mm00433287_ m1. mRNA expression levels were normalized to *B2m* expression, assay ID Mm00437762_m1, and presented as relative values.

### 2.15 | In-vitro assays

Human dermal fibroblasts (HdFbs) (Life Technologies C0135C) and human dermal microvascular endothelial cells (HdMVECs) (Life Technologies C01125PA) were purchased and used for in vitro assays. All assays were conducted in triplicate unless otherwise stated.

### 2.16 | Co-culture

Indirect co-culture experiments were performed using 6-well plates and 0.4 μM pore trans-well inserts. HdFb were seeded in the upper chamber and HdMVECs were seeded in the lower chamber. si-CXCL12 and scrambled-siRNA were purchased from Life Technologies. HMVECs were transfected using Lipofectamine RNAiMAX Reagent (Life Technologies) according to the manufacturer’s protocol before co-culture with HdFb in normoxia and hypoxia as previously described.^10^

### 2.17 | Proliferation

Human dermal fibroblasts were plated in 96-well cell-culture plates, 2500 cells/well, in 150 μL of medium with 1% FBS. After 24 h, fresh media with 1% FBS alone (control) or with varying concentrations of recombinant *Cxcl12* (25, 50, and 100 ng/mL) were added and after an additional 6 h Bromodeoxyuridine (BrdU) was added, and a cell proliferation assay was performed according to the manufacturer’s instructions (Roche Applied Sciences).

### 2.18 | Migration

Scratch assay was performed as previously described^24^ on HdFb cultured in 24-well plates with culture medium containing 1% FBS alone or with varying concentrations of recombinant *Cxcl12* (25 and 100 ng/mL).

### 2.19 | Survival

HdFb were cultured until 90% confluent in 24-well plates. They were then placed in culture medium containing 1% FBS for 24 h then cultured in medium containing 0.5% FBS alone (control), 100 ng/mL recombinant *Cxcl12*, 100 ng/mL recombinant *Cxcl12* + 10 μM U0126 (Cell Signaling Technology), or 100 ng/mL recombinant *Cxcl12* + 10 μM LY294002 (Cell Signaling Technology). After 72 h, images of 5 HPFs/well were captured and recorded under phase contrast microscopy, and manual cell counts were performed by a blinded observer.

### 2.20 | Microfluidic single-cell gene expression analysis

Gene lists were collected from a literature search. Single-cell reverse transcription and low cycle pre-amplification were performed as previously described.^25,26^ Wound lysate cell suspensions were sorted from *Tie2-Cre/Cxcl12^loxP/loxP^* and *Cxcl12^loxP/loxP^* transgenic mice as single progenitor cells into each well of a 96-well plate using a Becton Dickinson FACS Aria flow cytometer (Franklin Lakes, NJ) into 6 μL of lysis buffer and SUPERase-In RNAse inhibitor (Applied Biosystems, Foster City, CA) (*N* = 5 mice). Live/dead gating was performed based on DAPI exclusion. A total of 384 cells were analyzed in four 96-well arrays. Progenitor cells were defined as previously described. Reverse transcription and low cycle pre-amplification were performed using Cells Direct (Invitrogen) with Taqman assay primer sets (Applied Biosystems) as per the manufacturer’s specifications. Exon-spanning primers were used where possible to avoid amplification of genomic background. cDNA was loaded onto 96.96 Dynamic Arrays (Fluidigm, South San Francisco, CA) for qPCR amplification using Universal PCR Master Mix (Applied Biosystems) with a uniquely compiled Taqman assay primer set (Figure S2B) as previously described.^25^ Single-cell transcriptional data is available at GEO under accession number GSE146529.

### 2.21 | Statistical analysis

Data are illustrated as mean ± standard error of the mean (SEM). Data analysis was performed using Prism 9 (GraphPad, La Jolla, CA). For comparison between the two groups, Student’s *t*-tests (unpaired and two-tailed) were utilized. One- or two-way analysis of variance (ANOVA) was used for comparison of >2 groups. A *p*-value <.05 is considered statistically significant. Analysis of single-cell data was performed as described previously.^25–27^ Briefly, data from all samples were normalized relative to the pooled median expression for each gene and converted to base 2 logarithms. Absolute bounds (+/− 5 cycle thresholds from the median or 32-fold increases/decreases in expression) were set, and non-expressers were assigned to this floor. Clustergrams were then generated using hierarchical clustering (with a “complete” linkage function and Euclidean distance metric) to facilitate data visualization (MATLAB R2011b, MathWorks, Natick, MA).

To detect overlapping patterns within the single-cell transcriptional data, *k*-means clustering was employed using a standard Euclidean distance metric. Accordingly, each cell was assigned membership to a specific cluster as dictated by similarities in expression profiles (minimizing the within-cluster sum of square distances) in MATLAB. Optimally partitioned clusters were then sub-grouped using hierarchical clustering to facilitate visualization of data patterning.^37,38^ Non-parametric, two-sample Kolmogorov–Smirnov (K–S) tests were used to identify those genes with expression patterns that differed significantly between population clusters and/or groups, following Bonferroni correction for multiple samples using a strict cutoff of *p* < .05. For subgroup comparisons, the empirical distribution of cells from each cluster was evaluated against that of the remaining cells in the experiment. Ingenuity pathway analysis (IPA, Ingenuity Systems, Redwood City, CA) was used to construct transcriptome networks based on genes that were significantly increased within clusters (including both direct and indirect relationships).

## 3 | RESULTS

### 3.1 | Endothelial-specific CXCL12 does not regulate embryogenesis or vasculogenesis

To specifically assess the role of endothelial-specific CXCL12 during neovascularization, we engineered a floxed allele of *Cxcl12* (*Cxcl12 ^loxP/loxP^*) specific to our study (Figure 1A). *Rosa-CreER* and *Tie2-Cre* transgenes were utilized to generate tamoxifen-inducible global CXCL12 knockout (gKO) and endothelial-specific CXCL12 knockout (eKO) mice (Figure 1B), respectively.^11,12,28^ Floxed littermates negative for Cre expression (*Cxcl12^loxP/loxP^*) were utilized as our control group (WT) (Figure 1B). CXCL12 knockout progeny (*Cxcl12^loxP/loxP^*, *ROSA-CreER*^+/−^, *Tie2-Cre* ^+/−^) were viable, fertile, produced at expected Mendelian ratios, and showed no overt pathologic phenotype.

We confirmed DNA recombination upon tamoxifen administration with a subsequent >80% decrease in endothelial-specific *Cxcl12* mRNA and ensuing CXCL12 protein expression (Figure 1C–E). Endothelial-specific knockout of CXCL12 was additionally validated using immunostaining (Figure 1F). Prior work has demonstrated that stromal CXCL12 expression is essential throughout embryogenesis, cardiac development, hematopoiesis, and organ vascularization.^29–31^ Histologic analysis of cardiac, lung, gastrointestinal, and integument tissues was utilized to examine organ development and vascularization in eKO mice demonstrating physiologic morphology and vascular patterning (Figure S1A). This suggests that endothelial-specific CXCL12 does not have a primary role during organogenesis and vascular development.

*Tie2-Cre*-mediated deletion of *Cxcl12* has been extensively documented as being endothelial, non-hematopoietic (e.g., endothelial, perivascular stroma) cell-specific.^11,12^ To confirm that *Tie2-Cre*-mediated deletion of *Cxcl12* does not have deleterious effects on hematopoietic cells, we performed immunostaining for CD45, a nucleated hematopoietic cell-surface marker involved in immunologic activation and modulation,^32^ which demonstrated significant upregulation of CD45 in eKO compared to WT (Figure 1G,H). Further, to understand the direct impact that endothelial-specific CXCL12 has on neovascularization and angiogenesis, we performed immunostaining of eKO and WT mice tissue for von Willebrand factor (vWF), a glycoprotein marker expressed on endothelial cells.^33^ We observed a significant decrease in vWF expression in eKO mice compared to WT (Figure 1I,J). These findings demonstrate that endothelial-specific CXCL12 regulates the vascular density of the wound microenvironment during cutaneous wound healing without loss of hematopoietic cell recruitment or survival.

### 3.2 | Vascular, endothelial-specific CXCL12 critically regulates neovascularization and tissue repair

**FIGURE 2:**
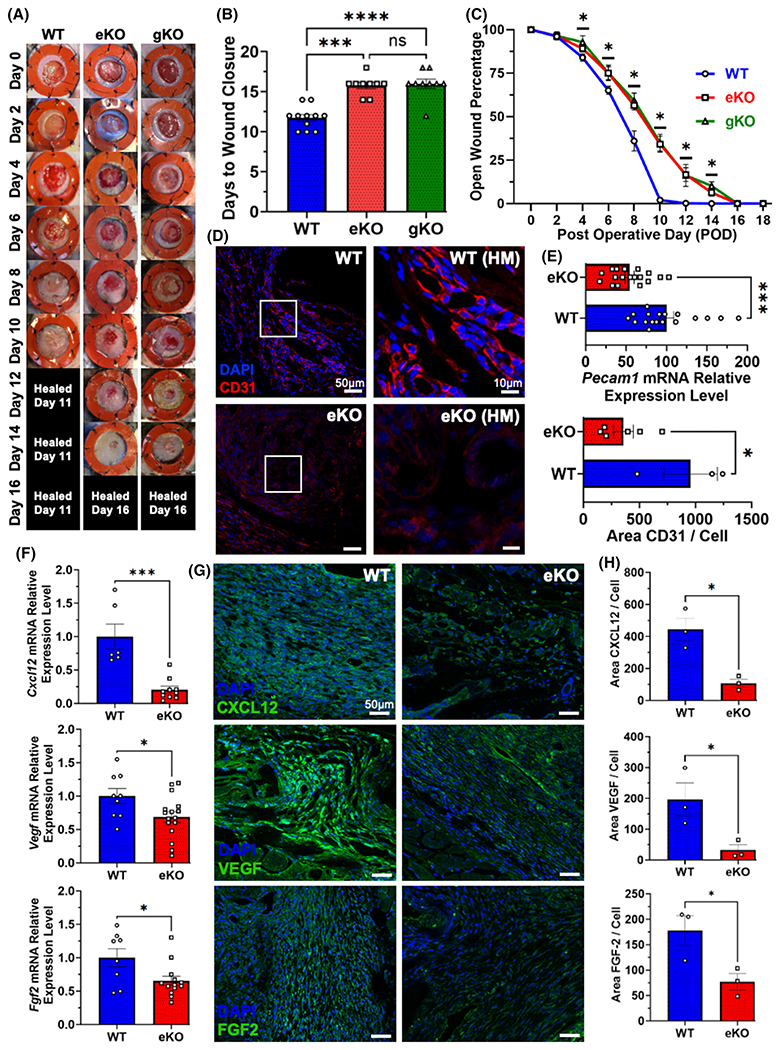
Wound healing relies on endothelial-specific CXCL12. (A) Gross pathologic photography of WT, eKO, and gKO representative wounds depicting delayed wound healing in eKO (*n* = 9) and gKO (*n* = 9) mice compared to WT (*n* = 11). (B, C) Similarly, eKO and gKO mice required 16 days to achieve wound closure compared to 11 days in WT (WT vs. eKO: ***p = .0001, WT vs. gKO: ****p = <0.0001, eKO vs. gKO: ns *p* = .9525). (D, E) Immunofluorescent staining, qRT-PCR, and immunofluorescent analysis of POD7 wound tissue for *Pecam1*/CD31 (red) marking vascular density (*p = .022, WT: *n* = 3, eKO: *n* = 6), (F) Decreased mRNA relative expression of *Cxcl12* (top), *Vegf* (middle), and *Fgf2* (bottom) in POD 7 eKO wound tissue compared to WT (*Cxcl12*: ***p = .0002, WT: *n* = 6, eKO: *n* = 10; *Vegf*: *p = .0298, WT: *n* = 9, eKO: *n* = 16; *Fgf2*: *p = .0227, WT: *n* = 8, eKO: *n* = 13). (G, H) Immunofluorescent staining and analysis denoting decreased CXCL12 (top, green), VEGF (middle, green), and FGF-2 (bottom, green) expression in eKO mice (CXCL12: *p = .0113, VEGF: *p = .0424, FGF-2: *p = .0393, WT: *n* = 3, eKO: *n* = 3). Statistical analysis was performed either using a two-way analysis of variance (ANOVA) with Tukey’s multiple comparison test (B, C) or an unpaired, two-tailed *t*-test (E, F, H). Each datapoint represents an independent wound (B, C, E, H) or independent measurement (F). All data are presented as mean ± SEM. Images (D, G) were obtained with a Zeiss LSM880 Inverted confocal microscope, magnification ×20, scale bar 50 μm, and 10 μm for high magnification (HM).

We then investigated the role of endothelial-specific CXCL12 in adult tissue repair using an excisional cutaneous injury model in eKO and gKO mice.^16^ On gross examination, we found that both eKO and gKO mice demonstrated delayed wound healing compared to WT (16 days vs. 11 days, respectively) (Figure 2A,B). The impairment in tissue repair in both eKO and gKO knockout groups was apparent on gross examination by postoperative day (POD) 4, as measured by the remaining wounded area (Figure 2C). Additionally, both the gKO and eKO groups had near identical healing times (achieving wound closure on POD 16), that were not statistically significantly different. The similarities in eKO and gKO wound healing (Figure 2A–C) suggest that endothelial cells are the critical CXCL12 source involved in neovascularization during tissue repair.

Microscopically, the most obvious difference in the healing tissue of eKO mice at POD 7 was a decreased vascular density demonstrated by immunostaining for CD31 (e.g., platelet endothelial cell adhesion molecule 1 [PECAM-1]), a transmembrane glycoprotein marker expressed by endothelial cells (Figure 2D,E).^34–36^ Decreased expression of CD31, in addition to the decrease in vWF expression in eKO mice (Figure 1I,J), further corroborated our aforementioned findings that endothelial-specific CXCL12 signaling modulates neovascularization and vascular density. Analysis of healing eKO wounded tissue at POD 7 compared to WT demonstrated reduced transcription and protein expression of CXCL12 in eKO mice. In addition, *Vegf* and *Fgf-2* mRNA transcription and VEGF and FGF-2 protein expression were diminished (Figure 2F–H), suggesting that CXCL12 signaling modulated other *Hif-1* regulated, angiogenic signaling pathways. To confirm the critical role of endothelial-specific CXCL12 signaling during neovascularization, we used a dorsal ischemic skin flap model.^10^ Upon gross examination of POD 10 ischemic flap tissue, we observed diminished tissue survival in eKO subjects (Figure S1B). Furthermore, to assess vascular density, we performed immunofluorescence staining for CD31 on ischemic flap tissue which demonstrated decreased expression in eKO mice (Figure S1C).

### 3.3 | Endothelial-specific CXCL12 regulates fibroblast gene expression, proliferation, and migration

**FIGURE 3:**
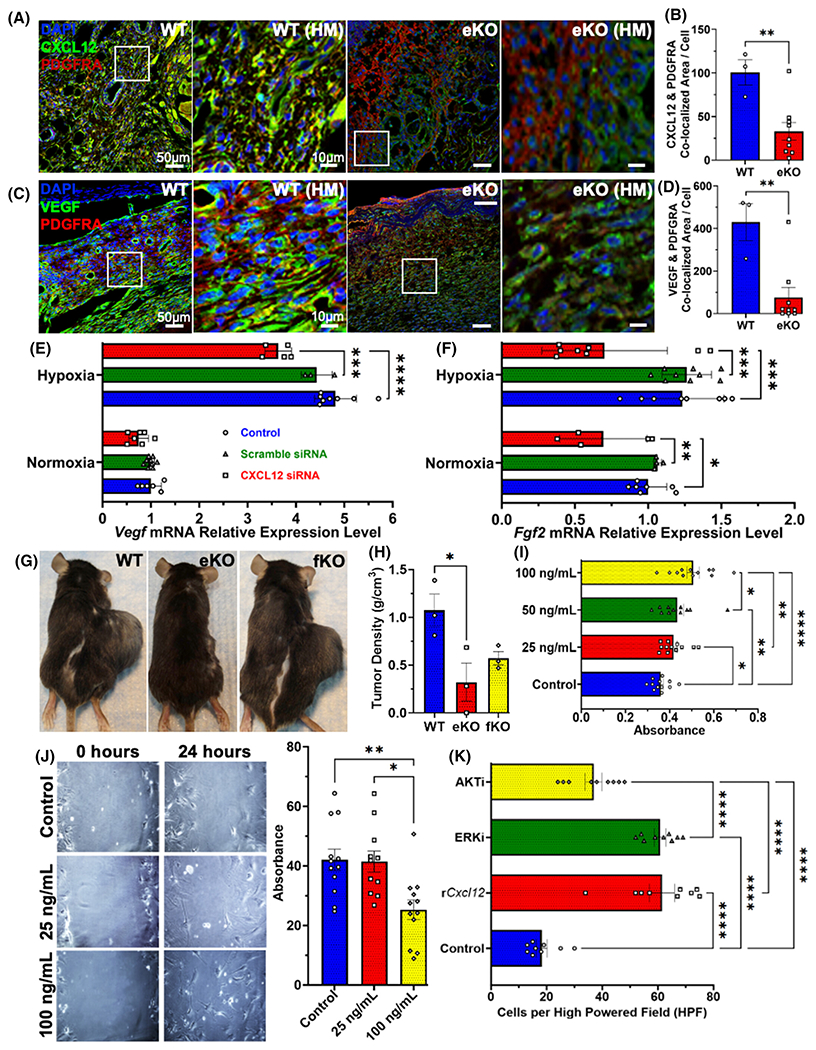
Endothelial-specific CXCL12 regulates neovascularization, stroma formation, and tumor progression. (A, B) Co-localized immunofluorescent staining on POD 7 wound tissue for PDGFRA, marking fibroblasts, and CXCL12 marking stromal-specific CXCL12 (**p = .0067, WT: *n* = 3, eKO: *n* = 9). (C, D) Immunofluorescent staining of eKO and WT POD 7 wound tissue for PDGFRA, marking fibroblasts, and VEGF marking angiogenesis (**p = .0039, WT: *n* = 3, eKO: *n* = 9). (E) Decreased fibroblast *Vegf* mRNA expression in the presence of decreased endothelial-specific *Cxcl12* expression under Normoxia (N) and Hypoxia (H) conditions (CXCL12 siRNA [H] vs. Control [H]: ****p < .0001, Scramble siRNA [H] vs. CXCL12 siRNA [H]: ***p = .0008, Control: *n* = 6 [N]/*n* = 8 [H], Scramble siRNA: *n* = 9 [N]/*n* = 3 [H]; CXCL12 siRNA: *n* = 7 [N]/*n* = 5 [H]). (F) Decreased fibroblast *Fgf2* mRNA expression in the presence of decreased endothelial-specific *Cxcl12* expression under Normoxia (N) and Hypoxia (H) conditions (CXCL12 siRNA [N] vs. Control [N]: *p = .0362, Scramble siRNA [N] vs. CXCL12 siRNA [N]: **p = .0065, CXCL12 siRNA [H] vs. Control [H]: ***p = .0006, Scramble siRNA [H] vs. CXCL12 siRNA [H]: ***p = .0003, Control: *n* = 7 [N]/*n* = 8 [H], Scramble siRNA: *n* = 4 [N]/*n* = 9 [H]; CXCL12 siRNA: *n* = 5 [N]/*n* = 8 [H]). (G, H) Reduced eKO tumor burden, as measured in density (g/cm^3^), compared to WT; (fKO = fibroblast-specific CXCL12 knockout) (*p = .0329, WT: *n* = 3, eKO: *n* = 3; fKO: *n* = 3). (I–K) Increased fibroblast proliferation, migratory capacity, and survival (mediated by PI3K/AKT signaling) in response to administration of incrementally increasing recombinant *Cxcl12* dosage (*p < .05, **p < .01 ***p < .001, ****p < .0001). Statistical analysis was performed either using an unpaired, two-tailed *t*-test (B, D), a one-way analysis of variance (ANOVA) (H), or a two-way ANOVA with Tukey’s multiple comparison test (E, F, I–K). All data are presented as mean ± SEM. Images (A, C) were obtained with a Zeiss LSM880 Inverted confocal microscope, magnification ×20, scale bar 50 μm, and 10 μm for high magnification (HM). Images (J) were obtained using a Zeiss Axioplan 2 fluorescence microscope, magnification ×20, scale bar 200 μm.

The establishment of new vascularized tissue requires a coordinated interplay between endothelial cells and fibroblasts.^37–39^ To characterize the downstream cellular and molecular effects of endothelial-specific CXCL12, we utilized immunofluorescent staining to examine the co-localization of fibroblasts using platelet-derived growth factor receptor alpha (e.g., PDGFRA), a common fibroblast marker,^40,41^ and CXCL12 in our eKO model. We found that knocking out endothelial-specific CXCL12 resulted in decreased fibroblast and CXCL12 co-localization (Figure 3A,B). This suggested that in the setting of an ischemic tissue injury, there is a paracrine intercommunication between endothelial cells and stromal fibroblasts driving tissue repair. In addition, we performed co-immunofluorescent staining for PDGFRA and vascular endothelial growth factor (e.g., VEGF), a well-defined marker for angiogenesis. We observed a significant decrease in co-localized PDGFRA and VEGF in eKO murine models compared to WT (Figure 3C,D). These findings demonstrate that endothelial-specific knockout of CXCL12 directly mitigates fibroblast CXCL12/VEGF expression, leading to downstream reductions in angiogenesis and neovascularization processes. Our in-vivo findings further illustrate the integral paracrine coordination between endothelial cells and fibroblasts that ultimately regulates neovascularization during tissue repair. Overall, endothelial cells play a key role in dictating pro-angiogenic fibroblast fates.

To explore the role of CXCL12 in endothelial–fibroblast crosstalk in-vitro, we used siRNA to target CXCL12 in human microvascular endothelial cells co-cultured with normal human dermal fibroblasts. Decreasing endothelial production of CXCL12 reduced fibroblast expression of VEGF and FGF-2 in response to hypoxia (Figure 3E,F). This data suggest that hypoxia-responsive fibroblast expression of VEGF and FGF-2, which stimulates endothelial cell proliferation, is in turn reliant on the endothelial expression of CXCL12 and provides a possible mechanism for our earlier immunostaining results in injured eKO skin (Figure 2F–H).

### 3.4 | Tumor growth is dependent on host vascular, endothelial-specific CXCL12 expression

Cancer cells, which typically exist in a setting of relative ischemia,^7^ appear to rely on the stromal microenvironment for tumor growth, angiogenesis, and invasion.^8^ In particular, melanoma has been shown to rely on its surrounding stroma.^42^ Typically, CXCL12 derived from cancer cells has been implicated in tumor pathogenesis, progression, and survival,^15^ including in B16 murine melanoma cells.^43^ To determine if host endothelial-specific CXCL12 played a role in tumor stroma formation and tumor progression, we transplanted B16 murine melanoma cells into control, endothelial-specific, and fibroblast-specific (*Col1a2-CreER* transgene) CXCL12 knockout (fKO) mice.^44,45^ The fKO model was chosen to investigate whether stromal cells themselves could produce CXCL12 in sufficient amounts to drive tumor progression, independent of endothelial cells or other circulatory sources. Tumor measurements demonstrated completely abrogated tumor growth in eKO mice (Figure 3G,H), suggesting a pivotal role for host endothelial-specific CXCL12 in tumor pathogenesis and progression.

Additionally, VEGF has been shown to impart drug resistance to tumor endothelial cells within the tumor stroma,^46^ indicating that paracrine signaling of endothelial-specific CXCL12 may induce tumor drug resistance. We sought to further examine this by treating fibroblasts with recombinant *Cxcl12*, which demonstrated amplified fibroblast proliferation and migratory capacity (Figure 3I,J). In addition, CXCL12 enhanced fibroblast survival in a low-nutrient environment (Figure 3K). Collectively, our findings indicate that host endothelial-specific CXCL12, in part, governs foundational elements of the tumor microenvironment, ultimately affecting the pathophysiology of carcinogenesis and progression.

### 3.5 | Endothelial-specific CXCL12 recruits a unique non-inflammatory circulating cell to injured tissue

**FIGURE 4:**
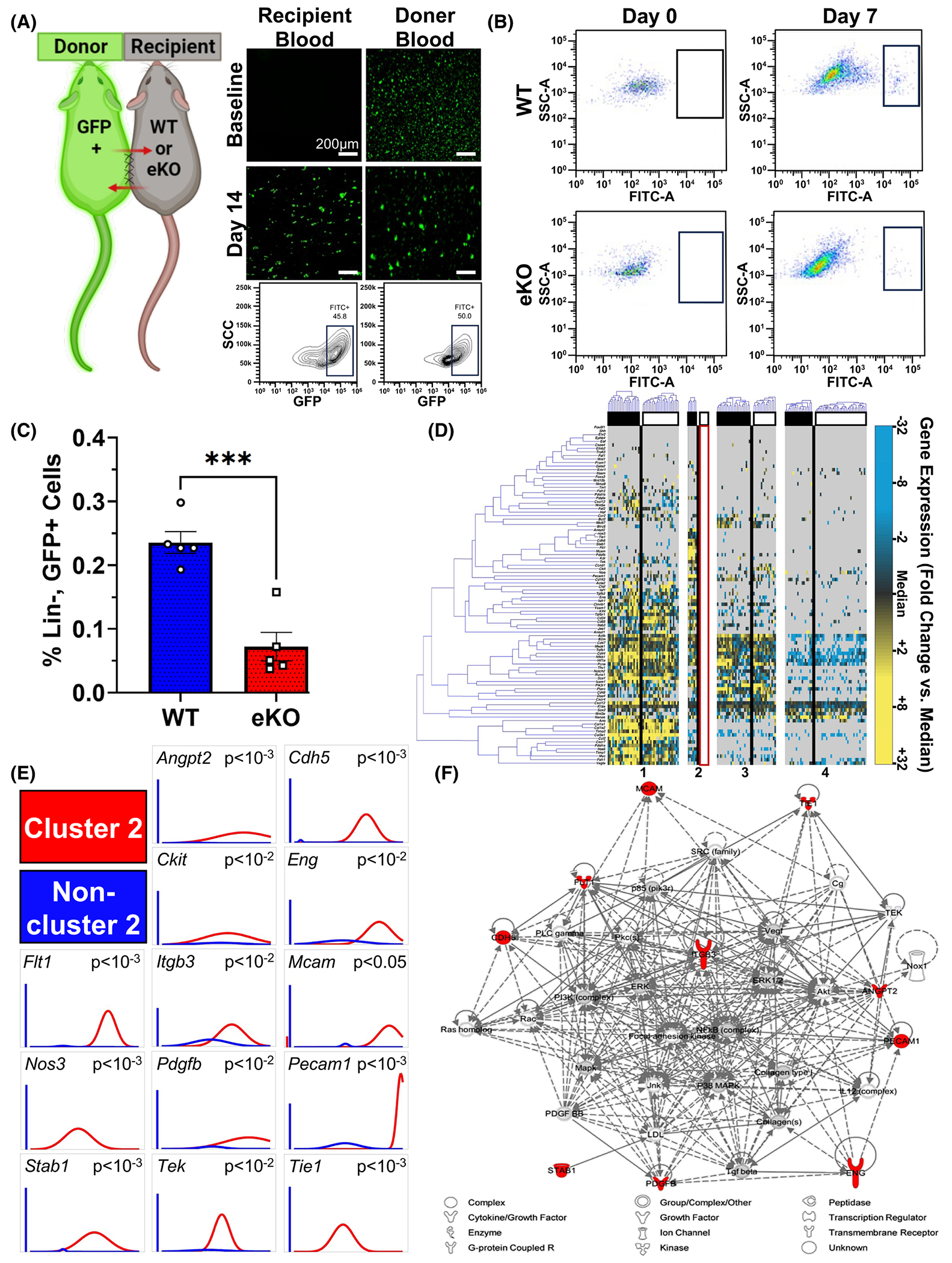
Endothelial-specific CXCL12 regulates the recruitment of progenitor cells in ischemic tissue. (A) Parabiosis schema and validation of successful cross-circulation. (B, C) FACS analysis illustrating reduced recruitment of non-inflammatory circulating cells (GFP^+^, Lin^−^) to parabiosed eKO wound conditions (***p = .0004, WT: *n* = 5, eKO: *n* = 5). (D) Partitional clustering revealed four transcriptionally distinct subpopulations of non-inflammatory progenitor cells throughout wounded tissue. The single-cell analysis demonstrated the absence of cluster 2 in eKO mice (white-headed columns) but preserved in WT (black-headed columns). (E) Marked absence of differentially expressed genes defining cell population in eKO wounds. Left bar for each panel represents fraction of cells that failed to amplify. (F) Top scoring Ingenuity Pathway Analysis (IPA)-constructed transcriptome network based on population, defining “seed” genes (E) (red) conditionally linking inferred genes (gray). Statistical analysis was performed using an unpaired, two-tailed *t*-test (C). Each datapoint represents an independent parabiosed WT or eKO subject (C). All data are presented as mean ± SEM. Images (A) were obtained using a Zeiss Axioplan 2 fluorescence microscope, magnification ×20, scale bar 200 μm.

Prior literature has established vascular endothelium as the central blood-tissue interface throughout the body.^47^ Furthermore, as endothelial cells are crucial for inflammatory cell recruitment,^48,49^ we sought to further characterize whether endothelial-specific CXCL12 has a role in neovascular-associated non-hematopoietic cell population recruitment.^50^ To investigate this further, we performed parabiosis on WT and/or eKO recipient mice to green fluorescent protein-positive (GFP^+^) donor mice (Figure 4A). After 3 weeks, immunofluorescent imaging and fluorescence-activated cell sorting (FACS) analysis of peripheral blood samples verified cross-circulation (Figure 4A). Excisional wounds were then induced to parabiosed eKO and WT dorsal skin. Wounded eKO and WT samples were collected on POD 7. FACS analysis demonstrated decreased circulating, undifferentiated progenitor cell (GFP^+^, Lin^−^) recruitment by eKO wounds (Figure 4B,C).

Furthermore, to characterize the circulating progenitor cells (GFP^+^, Lin^−^) differentially recruited to eKO and WT recipients during tissue repair, we utilized microfluidic technology to apply a massively parallel single-cell transcriptional analysis (SCA) (Figure S2). Partitional clustering revealed four transcriptionally distinct non-inflammatory, circulating progenitor cell subpopulations in WT wounded tissue and three subpopulations in eKO wounded tissue. Cluster 2 was completely absent, while another cluster was reduced in eKO subjects. The remaining two cell subpopulations were preserved across both WT and eKO (Figure 4D) (Figures S3 and S4). Further analysis of cluster 2, absent from injured eKO tissue, revealed progenitor cell-associated gene profiles, such as *Ckit* and *Mcam*, and vascular genes, such as *Pecam1*, *Flt1*, *Tie1*, and *Tek*. Additionally, the cluster 2 sub-population differentially expressed the cellular adhesion gene *Itgb3* (Figure 4E). Pathway analysis software was used to generate a transcriptional network utilizing those genes differentially expressed in this cluster as seed genes. “Inferred” genes included those known to be implicated in cell survival (*Erk1/2*, *Akt*, *Nfkb complex*), neovascularization (*Vegf*, *Pdgfb*), and extracellular matrix interactions (*Fak*). The overall associated biological functions of this network included cardiovascular system development and function, cellular movement, and cell morphology (Figure 4F). Collectively, this data indicates that endothelial-specific CXCL12 signaling regulates the recruitment of a unique non-inflammatory circulating cell to injured tissue.

## 4 | DISCUSSION

In this study, we describe the differential function of CXCL12 depending on its physiological context and tissue of origin. The endothelial expression does not regulate organ development or vascularization during embryogenesis, in contrast to stromal CXCL12 expression.^30,31^ However, endothelial-specific CXCL12 expression modulated the expression of hypoxia-responsive, angiogenic genes. Moreover, expression of endothelial-specific CXCL12 regulated angiogenic and neovascular responses throughout repair and regeneration following ischemic tissue damage as well as tumor pathogenesis and progression. Although the chemotactic effect of CXCL12 in the context of leukocytes has been investigated,^51^ its physiological function and cellular source in the context of injury had not been previously elucidated. We propose that a distinct population of circulating, non-inflammatory progenitor cells, originating from the bone marrow, are exclusively trafficked to injured tissue by CXCL12-mediated angiocrine signaling.

In the context of bone marrow cellular populations, *Tie2-Cre;Cxcl12* knockout is well documented as being endothelial-specific as it impacts non-hematopoietic cell lineages.^11,12^ CXCL12 expression is most significant in bone marrow-associated non-hematopoietic cells and only minorly expressed in bone marrow-associated hematopoietic cells.^11,12^ Prior literature has established that CXCL12 expression by hematopoietic bone marrow cells does not significantly contribute to the continuation or preservation of hematopoietic cell lineages.^11,12,52^ Our work, in culmination with prior literature, indicates that *Tie2-Cre*-mediated deletion of *Cxcl12* has specificity to non-hematopoietic cell lineages, implicating endothelial-specific CXCL12’s modulatory role in neovascularization vascular recruitment during the tissue repair process following ischemic tissue injury.

Finally, our findings extend previous work examining the relationship between tumor pathobiology and the mechanisms underlying tissue repair.^53^ Targeting the tumor microenvironment is an emerging paradigm in the management of resistant tumors. Our results demonstrate that host CXCL12 critically regulates the microenvironment independent of tumor-derived CXCL12, presenting a potential target for clinical therapy. However, it remains unclear why the selective inactivation of CXCL12 in endothelial cells has such profound effects on tumor growth despite the expression of CXCL12 by several other cell types, including tumor endothelial cells. Our findings suggest that endothelial-specific CXCL12 has a significant role in activating fibroblasts to stimulate downstream angiogenesis, and this endothelial-fibroblast crosstalk could also contribute to other disease states such as tumor growth. However, further investigation should be performed to more specifically interrogate the direct downstream molecular pathways influenced by endothelial-specific CXCL12, with a focus on angiogenesis and fibroblast behaviors. Our findings may have implications for the development of personalized oncological therapies, as understanding patient-specific biological responses to cancer may be more crucial than tumor profiling. While this study does not exhaustively explore the mechanisms underlying the similar effects of endothelial-specific CXCL12 on tumor growth and tissue repair, there is increasing evidence in the literature of the molecular and cellular similarities between wound healing and tumorigenesis.^54–56^ Further research parsing out potential differences are likely necessary to inform the development of clinical therapies.

## Supplementary Material

Supplementary materials

Additional supporting information can be found online in the Supporting Information section at the end of this article.

## Data Availability

All reported means and graphed data presented throughout the study are included in the article. Single-cell data are available at GEO, accession number GSE146529.

## Abbreviations:

μm: micrometer
ANOVA: analysis of variance
APLAC: administrative panel on laboratory animal care
BrdU: Bromodeoxyuridine
CD: cluster of differentiation
cDNA: complimentary DNA
cm: centimeter
CXCL12: C-X-C motif chemokine ligand 12
D: density
DAPI: 4′,6-diamidino-2-phenylindole
DNA: deoxyribonucleic acid
eKO: endothelial-specific CXCL12 knockout
FACS: florescence-activated cell sorting
FBS: fetal bovine serum
FGF-2: fibroblast growth factor 2
fKO: fibroblast-specific CXCL12 knockout
FLP: recombinase flippase
FRT: flippase recognition target
FSC: forward scatter
g: gram
GFP: green fluorescent protein
gKO: global CXCL12 knockout
H: height
H&E: hematoxylin and eosin
HdFbs: human dermal fibroblasts
HdMVECs: human dermal microvascular endothelial cells
HIF-1: hypoxia-inducible factor 1
HM: high magnification
HPF: high power field
IPA: ingenuity pathway analysis
L: length
M: mass
mL: milliliter
mm: millimeter
mRNA: messenger RNA
ng: nanogram
PBS: phosphate buffered saline
PCR: polymerase chain reaction
PDGFRA: platelet-derived growth factor receptor alpha
PECAM1: platelet endothelial cell adhesion molecule 1
PFA: paraformaldehyde
POD: post operative day
qRT-PCR: quantitative real time PCR
RBG: red blue green
rCXCL12: recombinant CXCL12
RNA: ribonucleic acid
SCC: systemic circulating cells
SDF-1: stromal cell-derived factor 1
SEM: standard error of the mean
siRNA: small interfering RNA
SSC: side scatter
V: volume
VEGF: vascular endothelial growth factor
vWF: von Willebrand factor
W: width
WT: wild type
