# Supplementary material for: Endothelial-specific CXCL12 regulates neovascularization during tissue repair and tumor progression
Source: FASEB J. Author manuscript; Available in PMC 2026 Apr 6. (PMC13051727; doi:10.1096/fj.202401307R)
Supplement: Supplementary materials [file NIHMS2147829-supplement-Supplementary_materials.docx]

**Supplemental Material:**

**Supplemental Figure 1.**

**
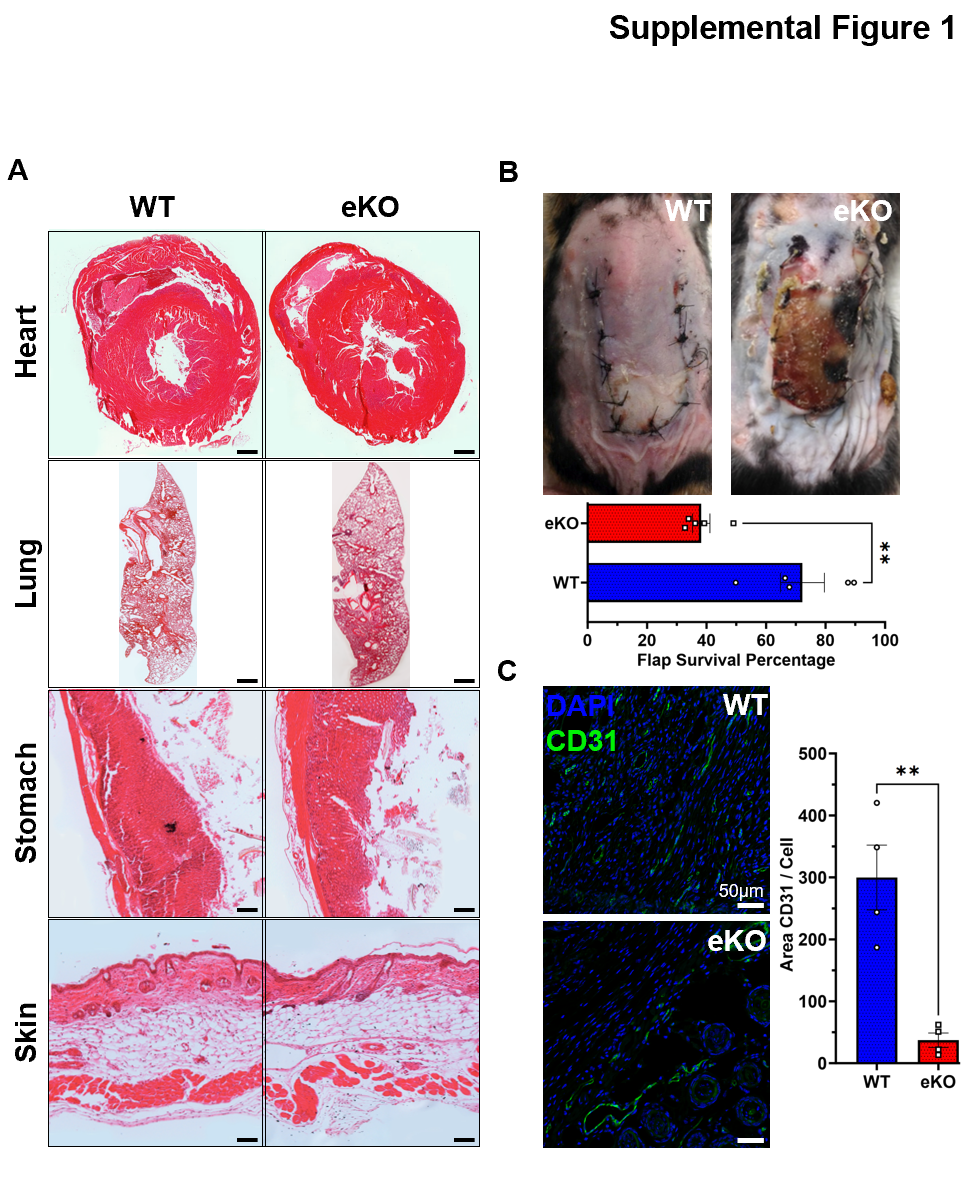
**

**Independent of morphogenesis, endothelial-specific CXCL12 modulates neovascularization during adult ischemic tissue repair.**

(**A**) Representative hematoxylin & eosin (H&E) staining of endothelial-specific CXCL12 knock-out (eKO) and wild type (WT) organs (e.g., heart, lung, stomach, and skin) exhibiting no endothelial-specific CXCL12 knock-out induced congenital anomalies. (**B**) Ischemic tissue flap model and analysis representing decreased gross flap survival in eKO mice at POD10 (**p*= 0.0027; WT: n = 5, eKO: n = 5). (**C**) Immunofluorescent staining and analysis of POD10 ischemic flap tissue for CD31 (green) marking vascular density (***p*= 0.0027; WT: n = 4 , eKO: n = 4). Statistical analysis was performed using an unpaired, two-tailed t-test (**B, C**). Each datapoint represents an independent wound (**B, C**). Images (**C**) obtained with a Zeiss LSM880 Inverted confocal microscope, magnification 20x, scale bar 50 µm.

**Supplemental Figure 2.**

**
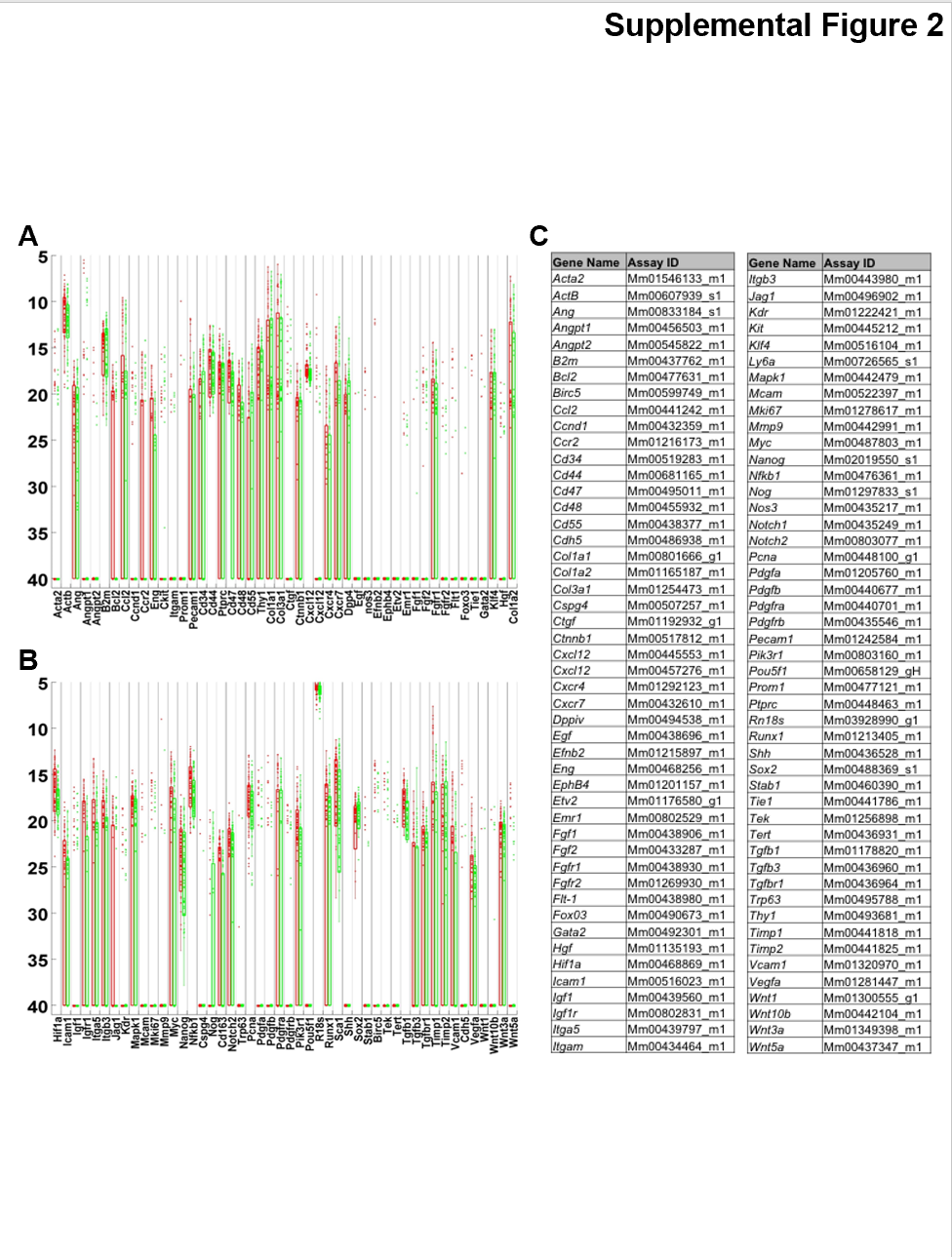
**

**Characterization of GFP^+^, Lin^-^ cellular genomic expression.**

(**A**) Whisker plot representment of qPCR cycle thresholds for GFP^+^, Lin^-^ gene expression from parabiosed WT (red) and parabiosed eKO (green). Single gene/cell qPCR reactions are characterized as individual points. Increased cycle threshold values correspond to decreased mRNA content and reactions that failed to achieve detection within 40 qPCR cycles were assigned values of 40. Boxes enclose the lower and upper quartiles, and whiskers delimit lowest/highest data points within 1.5 inter-quartile ranges (IQR) or lower/upper quartiles. (**B**) Taqman assays used to interrogate gene expression within GFP^+^, Lin^-^ cells.

**Supplemental Figure 3.**


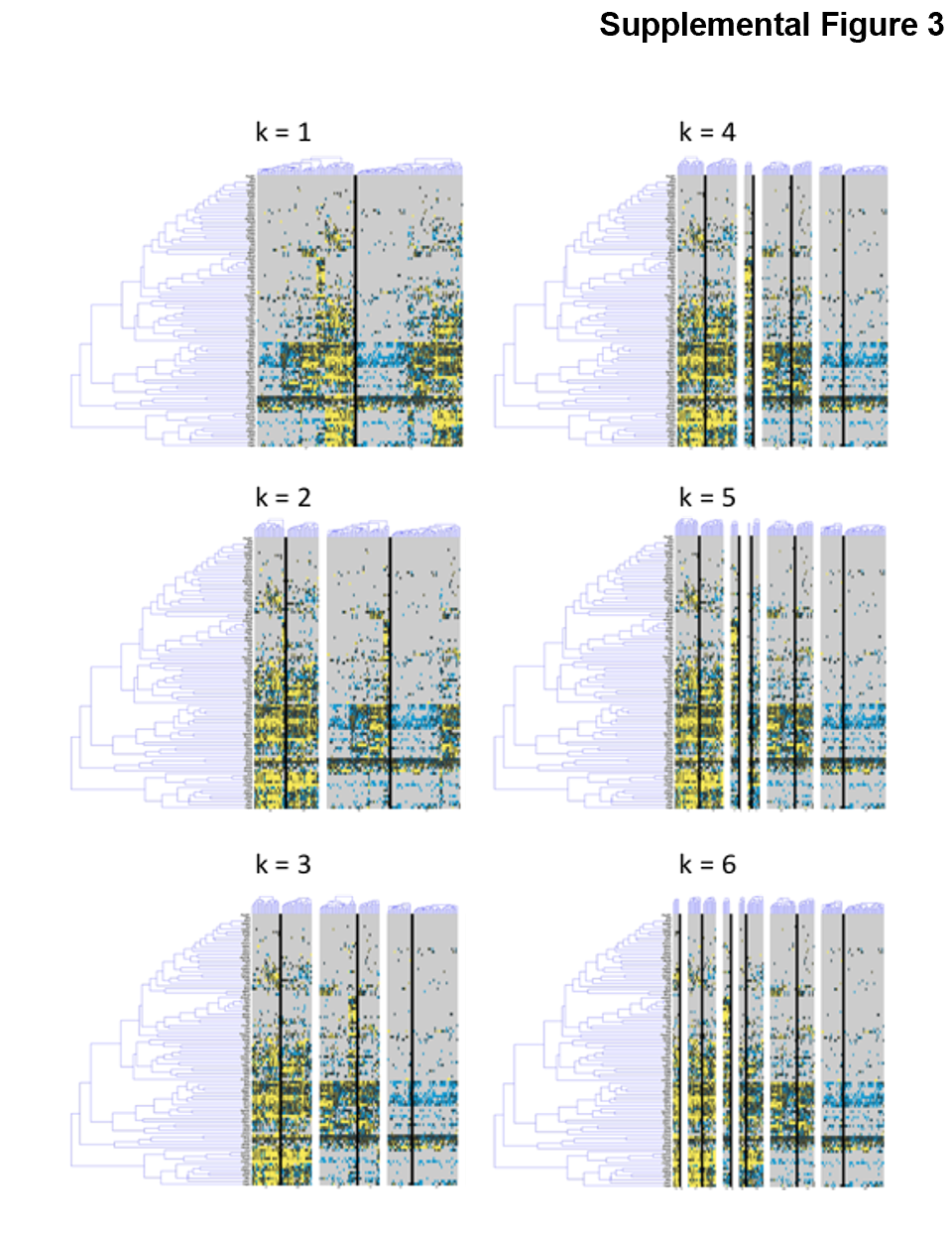


**Estimating the number of clusters in single-cell gene expression data.**

To estimate the appropriate number of subgroups that are present within our dataset, we visualized k=1 to k=6 clusters to estimate the ideal cluster number. The optimal number of clusters is designated as that which maximizes valid clusters without the presence of a “junk” cluster; this corresponded to four clusters in our dataset.

**Supplemental Figure 4.**


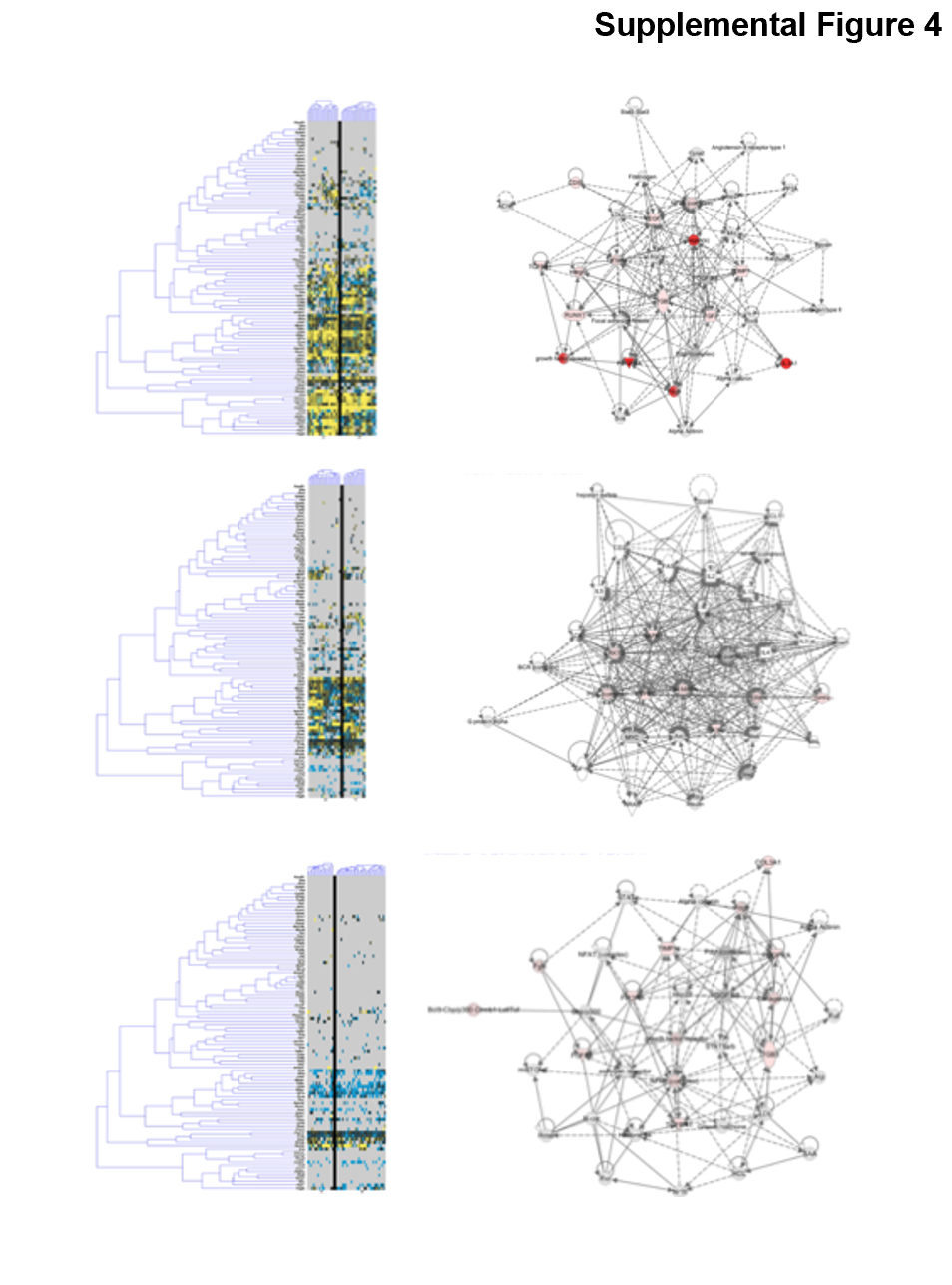


**Analysis of CXCL12’s role in modulation of progenitor cell transcriptome.**

(**A**) Single-cell transcriptome analysis depicting gene expression in WT (top, left), gKO (middle. left) and eKO (bottom, left) cells. (**B**) Top scoring Ingenuity Pathway Analysis (IPA)-constructed transcriptome network for the clusters present in WT and eKO wounds. These significant “seed” genes are colored in red to distinguish them from the remaining “inferred” genes in the network.
